# The plusses and minuses of DNA torsion

**DOI:** 10.7554/eLife.106351

**Published:** 2025-03-03

**Authors:** Steven Henikoff, David L Levens

**Affiliations:** 1 Basic Sciences Division, Fred Hutchinson Cancer Center Seattle United States; 2 Howard Hughes Medical Institute Chevy Chase United States; 3 Laboratory of Pathology, National Cancer Institute, National Institutes of Health Bethesda United States

**Keywords:** TMP-seq, transcription, torsional insulation, supercoiling, *S. cerevisiae*

## Abstract

A new method for mapping torsion provides insights into the ways that the genome responds to the torsion generated by RNA polymerase II.

**Related research article** Hall PM, Mayse LA, Bai L, Smolka MB, Pugh BF, Wang MD. 2025. High-resolution genome-wide maps reveal widespread presence of torsional insulation. *eLife* **14**:RP105675. doi: 10.7554/eLife.105675.

A key step in the transcription of DNA involves separating the two strands of the double helix so that an enzyme called RNA polymerase can move along one of the strands to produce messenger RNA. This separation produces a positive torsion in front of RNA polymerase, which would normally cause the strands ahead of the enzyme to rotate. However, if the strands are not free to rotate for some reason, the torsion will cause the DNA double helix to tighten up instead.

During transcription in eukaryotes, RNA polymerase II (PolII) encounters an array of nucleosomes just downstream of the transcriptional start site, each of which must be unwound so that the polymerase can keep moving along the strand it is transcribing. Because the DNA wrap around a nucleosome is left-handed, the positive torsion ahead of the polymerase will partially unwind the nucleosomes. However, the concerted action of all the different biomolecules involved in transcription – the polymerase, various enzymes called topoisomerases that can break and ligate strands of DNA, and a number of ATP-dependent remodelers – makes it difficult to study the role played by torsion in this process.

Among the methods that have been developed to map torsion is one that relies on trimethylpsoralen (TMP): this is a photosensitive molecule that inserts itself into the double helix and cross-links the two strands when it is illuminated with ultraviolet radiation (Bermúdez et al., 2010; Yang et al., 2014). In this method TMP is added to cells in culture and exposed to ultraviolet radiation, followed by fragmentation, DNA extraction and, finally, high-resolution mapping of the cross-linked sites. This TMP-seq approach can observe the difference in torsion between two systems – for example, between wild-type and mutant cells – but it cannot measure absolute torsion. Moreover, TMP is unable to access and cross-link DNA that is wrapped around nucleosomes, so TMP-seq can only be used to measure torsion in TMP-accessible DNA.

Now, in eLife, Michelle Wang (Cornell University) and co-workers – including Porter Hall (also of Cornell) as first author – report how they have been able to overcome some of the limitations associated with TMP-seq (Hall et al., 2025). After fixing budding yeast cells with formaldehyde and enzymatically removing the walls of these cells, the researchers used a restriction enzyme that cleaves double-stranded DNA at a four-base recognition site. This had the effect of relieving torsion throughout the genome. TMP-seq was then used to compare the torsion in cells that had been treated with the restriction enzyme and cells that had not been treated. In both cases, the very high resolution of TMP-seq revealed subtle but distinct differences between DNA that was accessible and DNA that was protected because it was wrapped around a nucleosome.

But what of the differences between the results obtained without the restriction enzyme and the results obtained when the DNA strands were free to rotate around one another because the restriction enzyme had been used? The researchers – who are based at Cornell and Penn State – found that negative torsion (that is, more TMP cross-links relative to the enzyme-treated sample) peaked just upstream of transcriptional start sites, and positive torsion (fewer cross-links) increased just downstream of transcription end sites (with increases in torsion correlating with levels of transcription and PolII occupancy). The differences in torsion were confined to regions of between 1 and 2 kilobase pairs around gene ends, indicating a short-range force driven by PolII. Negative torsion also peaked between divergent gene pairs, whereas positive torsion peaked between convergent genes, thus providing direct evidence for the twin-supercoiled domain model of transcription proposed almost 40 years ago (Liu and Wang, 1987).

These measurements of DNA torsion can test models for higher-order chromatin structures, and the observation of positive torsion peaks at paired contact sites of cohesin-bound DNA loops suggests that torsion has a role in regulating the 3D structure of the genome via cohesin (Jeppsson et al., 2024). It is also interesting to study divergent promoters that are close enough for the RNA polymerase at one to be affected by negative torsion from the RNA polymerase at the other, and Hall et al. found some 421 examples of this. The researchers also found 759 examples in which the divergent promoters were not coupled (a phenomenon known as torsional insulation) and there were sharp peaks of negative torsion just upstream of the transcription start sites. Hall et al. suggest that the region between two uncoupled promoters may be anchored to a cellular structure, and that this anchoring is strong enough to prevent rotation of the DNA during transcription. However, based on recent studies that explored local interactions in yeast (Mahendrawada et al., 2023) and long-range interactions in animals (Ke et al., 2024), we think that the torsional insulation observed by Hall et al. might also be explained by a model based on the local absorption of torsion by topological microdomains (Figure 1; Jha et al., 2022).

**Figure 1.**
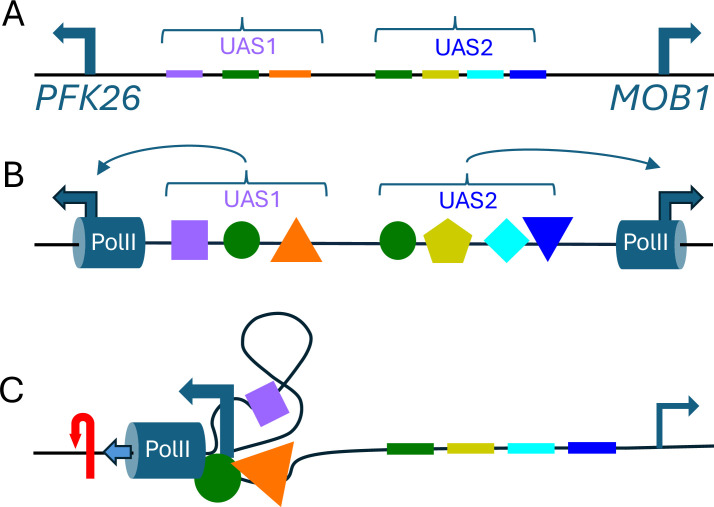
Torsional insulation and topological microdomains. (**A**) Hall et al. found numerous examples in which pairs of divergent promoters that were close to each other were not coupled to each other. Here we outline an alternative model to explain this phenomenon, which is known as torsional insulation, using the *PFK26-MOB1* region in yeast as an example: *PFK26* is activated by heat shock, but is not cell-cycle regulated; and *MOB1* is cell-cycle regulated, but is not activated by heat shock (Yan et al., 2015). The relatively short (less than 400 base pairs) region of the genome between these two genes contains binding sites (shown here in different colors) for seven different transcription factors, including insulating transcription factors (Abf1 and Reb1; Fourel et al., 2002) that prevent genes from being regulated by various genetic elements. (**B**) Multiple transcription factors (colored shapes) may bind to these sites; the transcription initiation machinery, including RNA Polymerase II (PolII; blue cylinders), also binds to the promoters (blue arrows) for each gene. (**C**) If some of the transcription factors interact with one another, and/or with the transcription initiation machinery (Wagh et al., 2025), the end result may be to restrict rotation or define a topological microdomain. During heat-shock in this model, PolII at *PFK26* would create positive torsion (red arrow) ahead of it and pump high levels of negative torsion backwards (to the right), only for this torsion to be absorbed by the transcription complex without reaching *MOB1* – and *vice-versa* for cell-cycle activation of *MOB1*. The transcription of just 10 base pairs introduces one supercoil (shown here by the plectoneme shape) into the topological microdomain and increases its superhelical density (Jha et al., 2022). In this model, the degree of insulation would evolve gradually over time with changes in the number and identity of the transcription- factor binding-sites between the genes. UAS: upstream activating sequence.

In summary, Hall et al. have developed a new method for mapping absolute torsion, and have used this method to distinguish torsion from DNA accessibility, to provide direct evidence for the twin-supercoiled domain model of transcription, and to explore torsional insulation in pairs of divergent promoters that are close to each other. The method could also be used to map the torsion in other situations, such as the torsion associated with chromatin loop structures. It will be interesting to determine how well features of the torsional landscape observed in budding yeast generalize throughout the eukaryotic kingdom.
